# Identification of Paroxysmal Nocturnal Hemoglobinuria-Type Bone Marrow Mast Cells

**DOI:** 10.7759/cureus.44919

**Published:** 2023-09-08

**Authors:** Süreyya Savaşan, Öner Özdemir, Manisha Gadgeel

**Affiliations:** 1 Pediatric Hematology/Oncology/Bone Marrow Transplantation, Children’s Hospital of Michigan, Detroit, USA; 2 Pediatrics, Central Michigan University, Mt. Pleasant, USA; 3 Oncology/Bone Marrow Transplantation, Barbara Ann Karmanos Cancer Center, Detroit, USA; 4 Hematology/Oncology Flow Cytometry Laboratory, Children’s Hospital of Michigan, Detroit, USA; 5 Pediatric Allergy and Immunology, Research and Training Hospital, Sakarya University Medical Faculty, Sakarya, TUR

**Keywords:** eculizumab, flow cytometry, bone marrow mast cells, children, aplastic anemia, paroxysmal nocturnal hemoglobinuria

## Abstract

Hematopoietic stem cells (HSCs) give rise to mast cells (MCs), and a relative increase in bone marrow (BM) MC is common in various BM failure (BMF) conditions. Paroxysmal nocturnal hemoglobinuria (PNH) is an acquired clonal HSC disorder, frequently associated with BMF, characterized by decreased expression of glycosylphosphatidylinositol membrane anchor for complement regulatory proteins. Eculizumab, a monoclonal antibody that blocks complement factor 5, successfully controls PNH symptoms. In this study, we, for the first time, demonstrated PNH-type culture-grown BM MC (c-BMMC) using flow cytometry in two BMF patients and monitored population size during eculizumab therapy. Further research may unravel the properties of PNH-type c-BMMC.

## Introduction

While hematopoietic stem cells (HSC) in bone marrow (BM) and peripheral blood (PB) can differentiate into several different blood and tissue cells, mesenchymal stem cells contribute to the hematopoietic microenvironment in BM and other tissues. Circulating basophils and tissue mast cells (MC) also originate from HSC. Acquired aplastic anemia (AAA) stands as a condition marked by defects in HSCs, ultimately leading to pancytopenia - a reduction in all three blood cell types. This depletion is triggered by an array of stressors, including radiation, cytotoxic agents, and infections. However, in the more commonly observed *idiopathic* cases, the prevailing mechanism is linked to an autoimmune response targeting HSCs. Whether there is an underlying HSC alteration facilitating the development of autoimmunity is not clear [1]. An interesting observation is the relatively increased presence of MC in the BM of hypoplastic/aplastic patients [2,3]. It has long been believed that increased MC is secondary to their relatively longer lifespan; however, there is no direct proof of this.

Paroxysmal nocturnal hemoglobinuria (PNH) is a rare clonal HSC disorder due to acquired PIGA mutations leading to decreased expression of glycosylphosphatidylinositol (GPI) membrane anchor and is frequently associated with AAA [4,5]. There is a growth advantage for PNH-type HSC over non-PNH counterparts in AAA, likely related to escaping autoimmune reactions. Detection of decreased expression of GPI-linked complement regulatory proteins, CD55 and CD59 on blood cells by flow cytometry is a diagnostic tool for PNH. Fluorescein aerolysin (FLAER) method has the advantage of detecting much smaller populations. Decreased or absent CD55 and CD59 can lead to intravascular hemolysis due to uncontrolled complement activation on red blood cells (RBCs), resulting in clinical and laboratory symptoms and findings. Activated complement factor 5-blocking monoclonal antibody, eculizumab, has been successful in controlling PNH symptoms [6].

To our knowledge, there are no studies demonstrating PNH-type MC. In this report, we present two cases in which PNH-type culture-grown BM MC (c-BMMC) were detected, and we investigate the impact of bone marrow recovery and PNH-directed therapy, including eculizumab, on the size of this population. This report was part of the Bone Marrow Failure (BMF) study investigating cellular components and interactions between them in patients with various types of BMF and was approved by the Institutional Review Board at Wayne State University. 

## Case presentation

Patient 1

A 17-year-old female patient with acquired severe aplastic anemia unresponsive to first-line immunosuppressive regimen was treated with high-dose cyclophosphamide, leading to some level of neutrophil recovery. She later developed PNH with signs of hemolysis, leading to the initiation of eculizumab therapy; BM cellularity was less than 5% at the time. She became transfusion-independent two months into eculizumab therapy, and BM reached 50% to 60% cellularity one year following PNH diagnosis.

Patient 2

An 11-year-old female patient presented with moderate thrombocytopenia, anemia with hemolysis, and 40% to 50% BM cellularity and was diagnosed with hypoplastic anemia and PNH. She was treated on eculizumab until she received allogeneic BM transplantation (BMT) four months after initial diagnosis.

Serial PB granulocyte, RBC, and c-BMMC PNH studies were performed during their clinical courses.

Methodology

PB Granulocyte and RBC PNH Staining

Granulocyte suspensions were prepared by performing ammonium chloride lysis followed by osmotic lysis of 0.5 mL of whole blood. The cells were then washed and re-suspended in 200 to 500 µL of phosphate-buffered saline (PBS) + 0.3% adult bovine serum + 0.025% ethylenediaminetetraacetic acid (EDTA) + azide buffer to obtain cell concentration between 1 × 10^6^ and 10 × 10^6^ cell/mL. To assess the presence and percentage of granulocytes by inspecting forward scatter (FS)/side scatter (SS), 50 µL of this suspension in 0.4 mL PBS was run on the flow cytometer. RBC suspensions were prepared by diluting 5 µL of whole blood to 0.5 mL PBS + 0.3% adult bovine serum + 0.025% EDTA + azide buffer, vortexing and transferring 50 to 200 µL of this suspension to a fresh tube containing 0.5 mL of the same buffer to match the RBC concentration to granulocyte cell concentration. Equal volumes of granulocyte and RBC suspension were then mixed for staining. Surface markers were then assessed for two-color phenotyping by incubating 50 µL of cell suspension with each of the following monoclonal antibody cocktails: CD15+CD235a-FITC/IgG1-PE, CD15+CD235a-FITC/CD55-PE, and CD15+CD235a-FITC/CD59-PE. Acquisition and analysis were performed on the Beckman Coulter Epics XL flow cytometer (Beckman Coulter, Brea, CA). Granulocyte and RBC were gated based on CD15-FITC log versus FS and CD235a-FITC log versus FS, respectively. CD55 and CD59 expressions on both granulocytes and RBC cells were analyzed for type I, type II, and type III PNH populations on single-color histogram.

MC Colony Development

Human MC was obtained by in vitro differentiation of BM-derived progenitor cells, as previously described [7]. In summary, BM mononuclear cells, isolated using Ficoll-Hypaque density gradient centrifugation, were suspended in a basic culture medium consisting of Iscove’s modified Dulbecco’s medium (IMDM) with 2-Mercaptoethanol (5 × 10-5 M) and 1% gentamycin. These cells were then cultured in methylcellulose medium (MethoCult H4236 Serum-Free, STEMCELL Technologies, Vancouver, BC, Canada) with the addition of stem cell factor (SCF, 200 ng/mL; Peprotech, Rocky Hill, NJ, USA), interleukin-6 (IL-6, 50 ng/mL), and IL-3 (5 ng/mL; eBioscience, San Diego, CA, USA). The cultures were plated at BM mononuclear cell density of 50,000 cells per well in six wells in a 24-well culture plate and incubated at 37 °C in 5% CO_2_. At week 2, serum-free methylcellulose medium supplemented with SCF (200 ng/mL) and IL-6 (50 ng/mL) was layered over the cultures. At week 4, cultures were layered with a basic culture medium supplemented with SCF (200 ng/mL), IL-6 (50 ng/mL), and 1% insulin-transferrin-selenium. The MC colonies were observed at week 6 and counted and characterized morphologically. Harvested colony cells with some macrophage contaminations were counted and checked for viability with Trypan blue staining.

c-BMMC PNH Staining

Flow cytometric immunophenotyping of the c-BMMC was performed with monoclonal antibodies, including CD45-FITC, CD117-PC5, CD34-PE, CD-2PE, CD25-FITC (Beckman Coulter, Brea, CA), CD55-PE (BD Bioscience, San Jose, CA), and CD59-PE (Invitrogen, Carlsbad, CA). Surface markers were assessed by three-color phenotyping with CD117-PC5 back-gating on the Beckman Coulter Epics XL flow cytometer. Mature MC was characterized by positive CD117 and negative CD2 and CD34 staining.

Changes in the MC colony/cell numbers and the frequency of PNH-type c-BMMC along with peripheral blood cells were studied at different times.

Results

**Figure 1:**
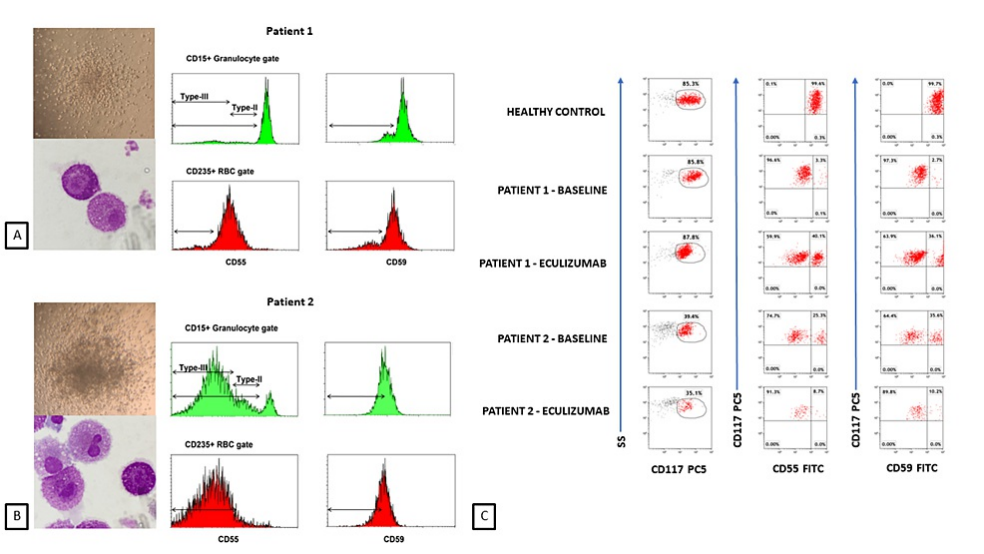
Microscopic image of MC colonies and c-BMMC and flow cytometric detection of PNH populations in peripheral blood granulocytes and red blood cells. Microscopic image of MC colonies (10×) and c-BMMC stained by Giemsa stain (100×) in (A) Patient 1 and (B) Patient 2. The level of granulation is different in the two cells.  (A and B) Flow cytometric detection of PNH populations in peripheral blood granulocytes and red blood cells characterized by decreased CD55 or CD59 and positive CD15 and CD235, respectively. Units in the x-axis represent the mean channel intensity of the respective conjugated monoclonal antibody binding and the y-axis represents the number of cells in monograms. (A-B) Type II and type III PNH cells are seen in CD55 granulocyte staining. (C) Detection of c-BMMC populations and their frequencies by positive CD117 staining using flow cytometry in two patients at different times. CD117-positive cells were first identified using SS and CD117 dot-blots. Then, gated CD117-positive cells stained with CD55 and CD59 identified PNH-type c-BMMC characterized by decreased CD55 or CD59 expression. (C) Units in the x- and y-axis represent the mean channel intensity of the respective conjugated monoclonal antibody binding in all dot-blots. c-BMMC, culture-grown bone marrow mast cells; MC, mast cell; PNH, paroxysmal nocturnal hemoglobinuria; SS, side scatter

Different numbers and sizes of c-BMMC colonies were observed and harvested colony cells displayed morphologic characteristics of MC with variable cytoplasmic granulation patterns (Figures 1A-1B). PNH-type peripheral blood granulocyte and RBC and c-BMMC were detected with varying PNH population sizes (Figures 1A-1C).

As the two cases have differences in their disease states and treatment approaches directed at BMF and duration of therapeutic interventions, PNH-type c-BMMC population sizes have differed while the patients were also on eculizumab therapy.

**Figure 2:**
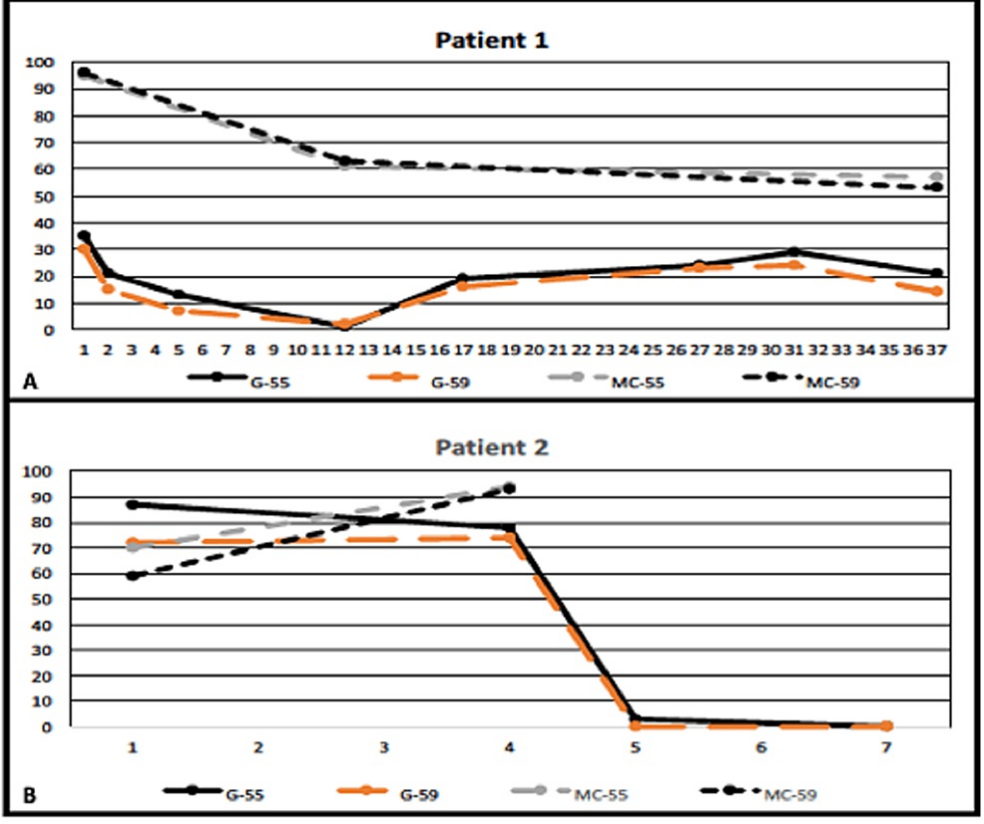
Correlation between PNH-type granulocytes and c-BMMC along with relative decrease in PNH-type hematopoiesis during eculizumab therapy. A positive correlation between PNH-type granulocytes and c-BMMC was observed due to bone marrow recovery along with a relative decrease in PNH-type hematopoiesis during eculizumab therapy in (A) Patient 1. Initial short follow-up period before BMT, the relative PNH-type c-BMMC population has increased likely secondary to an ongoing growth advantage of PNH-type hematopoiesis as no bone marrow failure-directed treatment is given in (B) Patient 2. Then, the PNH-type c-BMMC population completely disappeared following successful BMT in the second case. G-55 stands for *percentage of PNH-type granulocytes by low CD55 staining*, G-59 *percentage of PNH-type granulocytes by low CD59 staining*, MC-55 *percentage of PNH-type MC by low CD55 staining*, and MC-59 *percentage of PNH-type MC by low CD55 staining*. Units in the x-axis represent time in months, and the y-axis represents the percentage of PNH-type cells in the tested population. BMT, bone marrow transplantation; MC, mast cell; c-BMMC, culture-grown bone marrow mast cells; PNH, paroxysmal nocturnal hemoglobinuria

While in vitro MC colonies and harvested colony cell numbers increased 11 months into AAA-directed and eculizumab therapies paralleling overall BM cellular recovery, the relative PNH-type c-BMMC population decreased during that time and remained stable afterward in Patient 1 (Figure 1C). Interestingly, changes in PNH-type c-BMMC and granulocyte PNH relative clone size paralleled each other in the long term (Figure 2A).

However, in the second case, which had acquired hypoplastic anemia rather than AAA, c-BMMC colonies and harvested cell numbers dropped over three months likely to be related to ongoing anti-HSC immune reaction and consequent decrease in MC production. However, the relative PNH-type c-BMMC population rose before BMT while the patient was receiving eculizumab therapy (Figure 2B). This is likely to be due to the growth advantage for PNH-type HSC compared with the rest of the HSC population under proapoptotic stress through anti-HSC immune reaction [8]. Our findings point to an inverse relationship between normal HSC recovery and a decline in relative PNH-type c-BMMC population as was observed in the first case with long-term follow-up (Figure 2A). Whether eculizumab therapy contributed to a change in PNH-type peripheral blood granulocyte, RBC, or c-BMMC population sizes in this patient remains unknown. Studies with a larger number of cases and with longer follow-up periods may shed light on this question.

## Discussion

To our knowledge, this is the first report of human PNH-type MC obtained from c-BMMC in patients with PNH and the change in the population during BMF and PNH-directed therapies. In a single report, it was noted that GATA1-Cre-mediated PIGA inactivation resulted in culture-grown MC in mice that lacked FLARE binding [9]. There are cell membrane microdomains called lipid rafts, which are rich in cholesterol, sphingolipids, GPI-anchored proteins, and receptors. There is some evidence that lipid rafts play a role in MC endocytosis and degranulation [10]. Therefore, there is a possibility of PNH-type MC having functional differences from normal MC due to missing GPI structure and GPI-linked specific membrane proteins. Furthermore, there are up to 150 GPI-linked proteins in different cells, several of which are expressed on MC, and thus, investigations into PNH-type MC could help characterize their functions with potential clinical implications [11]. Studies investigating this possibility are underway.

## Conclusions

Human PNH-type c-BMMC can be detected in patients with PNH. Exploring the potential functional differences that may exist in these cells might provide useful information that can be exploited in allergic and inflammatory conditions where MC plays an important role.
